# Doxycycline directly targets PAR1 to suppress tumor progression

**DOI:** 10.18632/oncotarget.15166

**Published:** 2017-02-07

**Authors:** Weilong Zhong, Shuang Chen, Qiang Zhang, Ting Xiao, Yuan Qin, Ju Gu, Bo Sun, Yanrong Liu, Xiangyan Jing, Xuejiao Hu, Peng Zhang, Honggang Zhou, Tao Sun, Cheng Yang

**Affiliations:** ^1^ State Key Laboratory of Medicinal Chemical Biology and College of Pharmacy, Nankai University, Tianjin, 300000, China; ^2^ Tianjin Key Laboratory of Molecular Drug Research, Tianjin International Joint Academy of Biomedicine, Tianjin, 300000, China

**Keywords:** tumor, molecular target, GPCR, tumor progression, antibotics

## Abstract

Doxycycline have been reported to exert anti-cancer activity and have been assessed as anti-cancer agents in clinical trials. However, the direct targets of doxycycline in cancer cells remain unclear. In this study, we used a chemical proteomics approach to identify the Protease-activated receptor 1 (PAR1) as a specific target of inhibition of doxycycline. Binding assays and single-molecule imaging assays were performed to confirm the inhibition of doxycycline to PAR1. The effect of doxycycline on multi-omics and cell functions were assessed based on a PAR1/thrombin model. Molecular docking and molecular dynamic simulations revealed that doxycycline interacts with key amino acids in PAR1. Mutation of PAR1 further confirmed the computation-based results. Moreover, doxycycline provides highly selective inhibition of PAR1 signaling in tumors *in vitro* and *in vivo*. Using pathological clinical samples co-stained for doxycycline and PAR1, it was found that doxycycline fluorescence intensity and PAR1 expression shown a clear positive correlation. Thus, doxycycline may be a useful targeted anti-cancer drug that should be further investigated in clinical trials.

## INTRODUCTION

The anti-tumor activity of tetracyclines has been verified in many tumor types, and doxycycline has been subjected to evaluation in clinical trials [1–3]. Doxycycline has been shown to exert multipotent drug activity inhibit angiogenesis, metastasis, ribosome activity and mitochondrial function [4–7]. However, the anti-tumor functions of doxycycline and the proteins that mediate these molecular effects remain unknown.

Protease-activated receptor 1 (PAR1) is a member of the Guanosine-binding protein coupled receptor family, which has been implicated in metastatic and invasive processes associated with cancers as well as vascular biology and tissue remodeling [8]. A study by Kuliopulos et al. showed evidence for a role for PAR1 as an MMP-1 receptor that promotes invasion and tumorigenesis in breast cancers [9]. Here, we used a chemical proteomics approach involving covalent modification to identify PAR1 as a doxycycline-specific target protein. Molecular docking results and molecular dynamic (MD) simulations also confirmed the interaction between doxycycline and PAR1. This prediction was validated by biomolecular interaction analysis (Biacore) and mutational analyses. To verify the inhibitory capacity of doxycycline toward cancer cells via PAR1, multi-omics was used to identify critical pathways inhibited by doxycycline, and the results of a multivariate analysis strongly suggested that doxycycline inhibits PAR1 signaling pathways. Additionally, doxycycline selectively inhibited the progression of various types of cancer associated with high PAR1 expression both *in vitro* and *in vivo*.

## RESULTS

### Design, synthesis, activity assay and target validation of doxycycline probes

**Figure 1:**
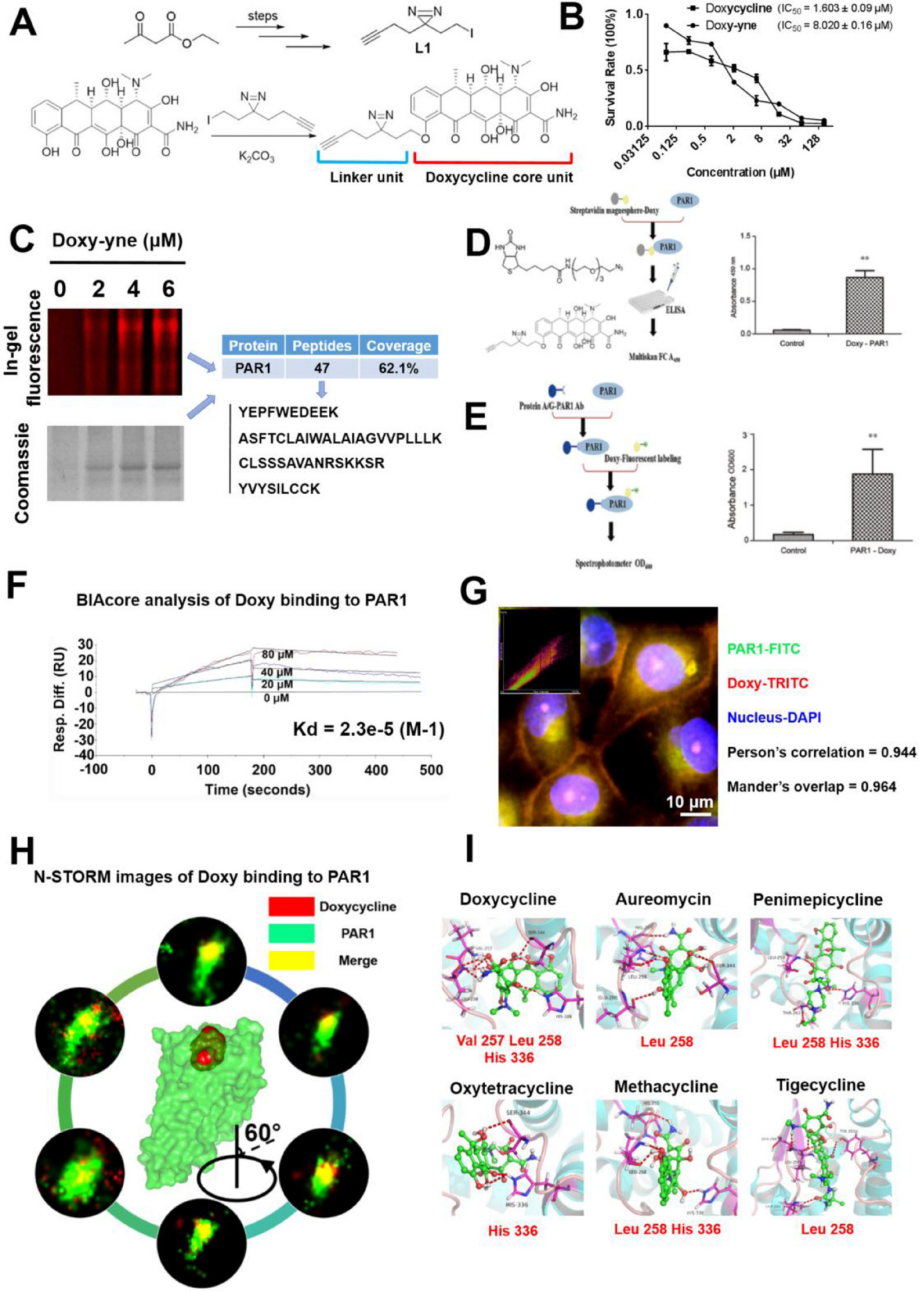
Design of the doxycycline probe and confirmation of doxycycline binding to PAR1 (**A**) Structure of the doxycycline probe used in this study. A portion of the doxycycline core unit and linker unit. (**B**) IC_50_ plots of doxycycline and doxycycline probe, as determined by the MTT assay. (**C**) Doxy-yne labeling of live cells and PAR1 pull-down with confirmation by MS. (**D**) Positive pull-down assays showing that the fluorescence of PAR1-Doxy was 100-fold greater than that of the control sample. (**E**) Reverse pull-down assays showing that the binding of doxycycline to PAR1 was 30-fold greater than that of the control sample. (**F**) Biacore analysis of doxycycline binding to PAR1 showing that doxycycline was captured by PAR1 with a *K*_d_ = 23 μM. (**G**) Confocal immunofluorescence imaging was performed to visualize PAR1 and doxycycline. PAR1 and doxycycline co-localize, with a Pearson’s correlation coefficient of 0.944 and a Mander’s overlap of 0.964. (**H**) N-STORM images of doxycycline bound to PAR1. These results show single molecules of doxycycline bound to PAR1, and 360-degree rotation reveals the combination image. (**I**) Binding mode of tetracyclines docked in the active site of PAR1. These results show that the entire tetracycline molecule occupies the binding pocket of PAR1 and that doxycycline demonstrates the strongest binding affinity and the lowest binding energy. Every experiment was repeated three times. The error bars represent the standard deviation (*P < 0.05, ^*^P < 0.01). Data are represented as mean ± SEM. See also Supplementary Figure 1.

As presented in Figure 1A, the doxycycline probe (doxy-yne) was designed using two essential structural components, the core unit (retains compound activity) and the linker unit (covalently binds to the target protein) [10, 11]. The synthesis of doxy-yne is shown in Figure 1A, and details are provided in Supplementary Figure 1. The linker unit (L1) was synthesized as previously described, after which L1 was reacted with doxycycline to complete the preparation of doxy-yne. To demonstrate the feasibility for the application and activity of doxy-yne, an *in vitro* inhibition assay was performed using A549 cell lines with an IC_50_ value of 8.02 μM, which is similar to doxycycline (Figure 1B). The results indicated that the inhibitory effect of doxy-yne was retained.

Based on SDS-PAGE and LC-MS/MS results, we confirmed PAR1 as a targeting protein with doxy-yne (Figure 1C). To confirm PAR1 as a target protein of doxycycline, forward and reverse doxy-yne pull-down assays were conducted (Figure 1D and Figure 1E). In the forward pull-down assay, the fluorescence values were approximately 100 times higher than those of the control sample (Figure 1D). Similarly, the experimental group displayed 30-fold greater fluorescence intensity in the reverse pull-down assay (Figure 1E). The Biacore results for four different concentrations of doxycycline yielded binding kinetics of *K*_d_ = 23 μM. The binding kinetics and *K*_d_ value strongly suggest binding between doxycycline and PAR1 [12]. Confocal immunofluorescence imaging revealed a high proportion of PAR1 and doxycycline probe co-localization, with a Pearson’s correlation coefficient of 0.944 and a Mander’s overlap of 0.964 (Figure 1G). Using a Super-Resolution Microscope System (N-STORM), we observed doxycycline co-localization with PAR1 in living cells on a nanoscale level [13]. Single-molecule imaging and docking results further confirmed the binding of doxycycline to the active site of PAR1 (Figure 1H and 1I). All tetracyclines were computationally docked with the active site of PAR1, and the models exhibited similar binding modes (Supplementary Table 1). Molecular docking analysis revealed the key amino acids that participate in the interaction between PAR1 and tetracyclines.

### Doxycycline specifically inhibits PAR1 through physical and functional binding

**Figure 2:**
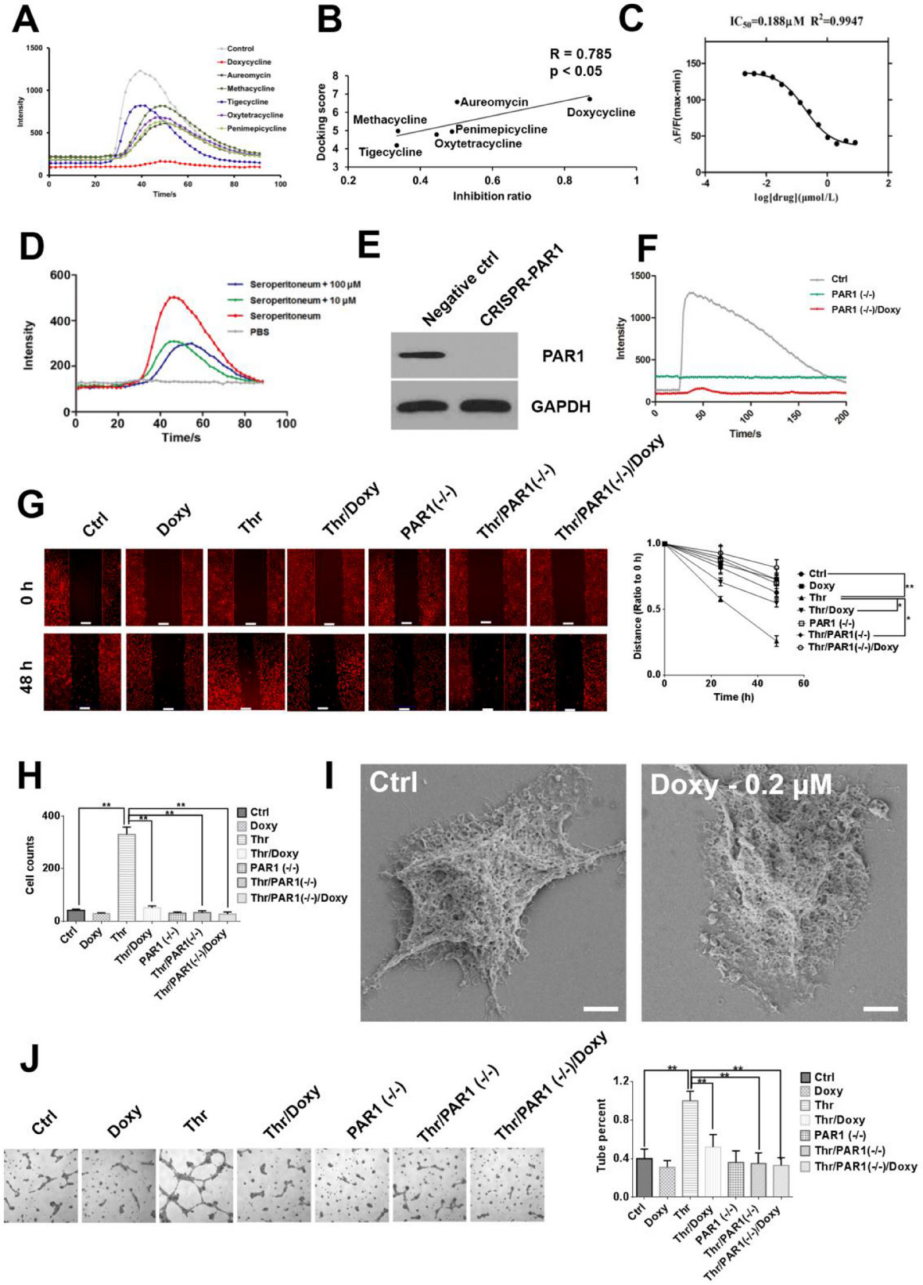
Doxycycline inhibits PAR1 and effectively decreases cancer cell malignancy (**A**) Calcium influx signals in HEK-293-PAR1 cells treated with different tetracyclines (1 μM). All tetracyclines displayed inhibitory effects, among which doxycycline had the strongest inhibitory effect. (**B**) Regression analysis of the inhibition ratio and docking scores. The correlation coefficient between these two indexes was as high as 0.785 (*P* < 0.05). (**C**) Doxycycline inhibits calcium flow signals in a dose-dependent manner. (**D**) Doxycycline partially inhibits calcium flow signals stimulated by seroperitoneum with 10 and 100 μM. These results indicate that tetracyclines do not directly chelate calcium ions but rather inhibit PAR1 activity. (**E**, **F**) PAR1 knockdown by CRISPR and abrogates the sensitivity of cells to thrombin. (**G**) Doxycycline inhibits cell migration induced by thrombin under 1.6 μM in 48 h. (**H**) The cells were treated with doxycycline with 24 h under 1.6 μM and invasion ability were decreased. Invasion ability of PAR1(−/−) cells were decreased compared with normal cells. (**I**) Cells were treated with 0.2 μM doxycycline for 24 h. Scanning electron microscopy revealed a reduction in lamellipodia and filopodia compared with the control group. (**J**) Tube formation in thrombin-stimulated cells, doxycycline inhibit pipe formation ability with 1.6 μM. Results were obtained from three independent experiments, each performed in triplicate, and the error bars represent the standard deviation (*P < 0.05, ^*^P < 0.01). Data are represented as mean ± SEM. See also Supplementary Figure 2.

PAR1 activation upon thrombin binding can induce calcium influx. Therefore, a calcium flow inhibitory assay was performed to measure the inhibition of calcium signaling stimulated by PAR1 [14]. Figure 2A shows that all 6 tetracyclines efficiently inhibited calcium signaling stimulated by thrombin, and doxycycline exhibited the strongest inhibitory activity. Computational results demonstrated a strong correlation between the docking scores and the corresponding inhibitory assay results (Figure 2B). Doxycycline also inhibited calcium-dependent signaling in a dose-dependent manner (Figure 2C).

To confirm that doxycycline inhibition of calcium signaling was not caused directly by the chelation of calcium ions, total inflammatory factors were used to activate cellular calcium signals. If doxycycline treatment results in calcium ion chelation, then it would observe no difference between the inhibitory effects on calcium signals caused by PAR1 and inflammatory factors. However, whereas doxycycline specifically inhibited calcium signaling caused by PAR1, it incompletely inhibited calcium signaling induced by general inflammatory factors, which includes thrombin (Figure 2D). These results indicate that tetracyclines do not function through the chelation of calcium ions but specifically inhibit PAR1-dependent calcium influx. When the PAR1 was knock down using CRISPR, the level of PAR1 expression decreased, and the sensitivity of the cells to thrombin was lost [15]. Doxycycline showed no inhibitory effect on PAR1-negative cells (Figure 2E and 2F). Doxycycline treatment also specifically inhibited thrombin-induced tumor cell migration in control cells to a greater extent than PAR1 low-expressing cells or PAR1-negative cells (Figure 2G and 2H and Supplementary Figure 2). Cells treated with doxycycline undergo significant changes in morphology, including pseudopod disappearance and cell shrinking, as detected by SEM (Figure 2I) [16]. In invasion assays, compared with the control group, the invasion ability increased significantly after the addition of thrombin. Additionally, doxycycline significantly inhibited thrombin/PAR1 cells compared with control cells but showed no effect on PAR1-negative cells. Furthermore, The effects of doxycycline on the vascular mimicry induced by PAR1 was examined. The tube formation rate was extremely high in the thrombin treatment group compared with the control group, and doxycycline significantly inhibited tube formation specifically via PAR1 inhibition (Figure 2J).

### Doxycycline occupies the active site and prevents “tethered agonist” activation of PAR1

**Figure 3:**
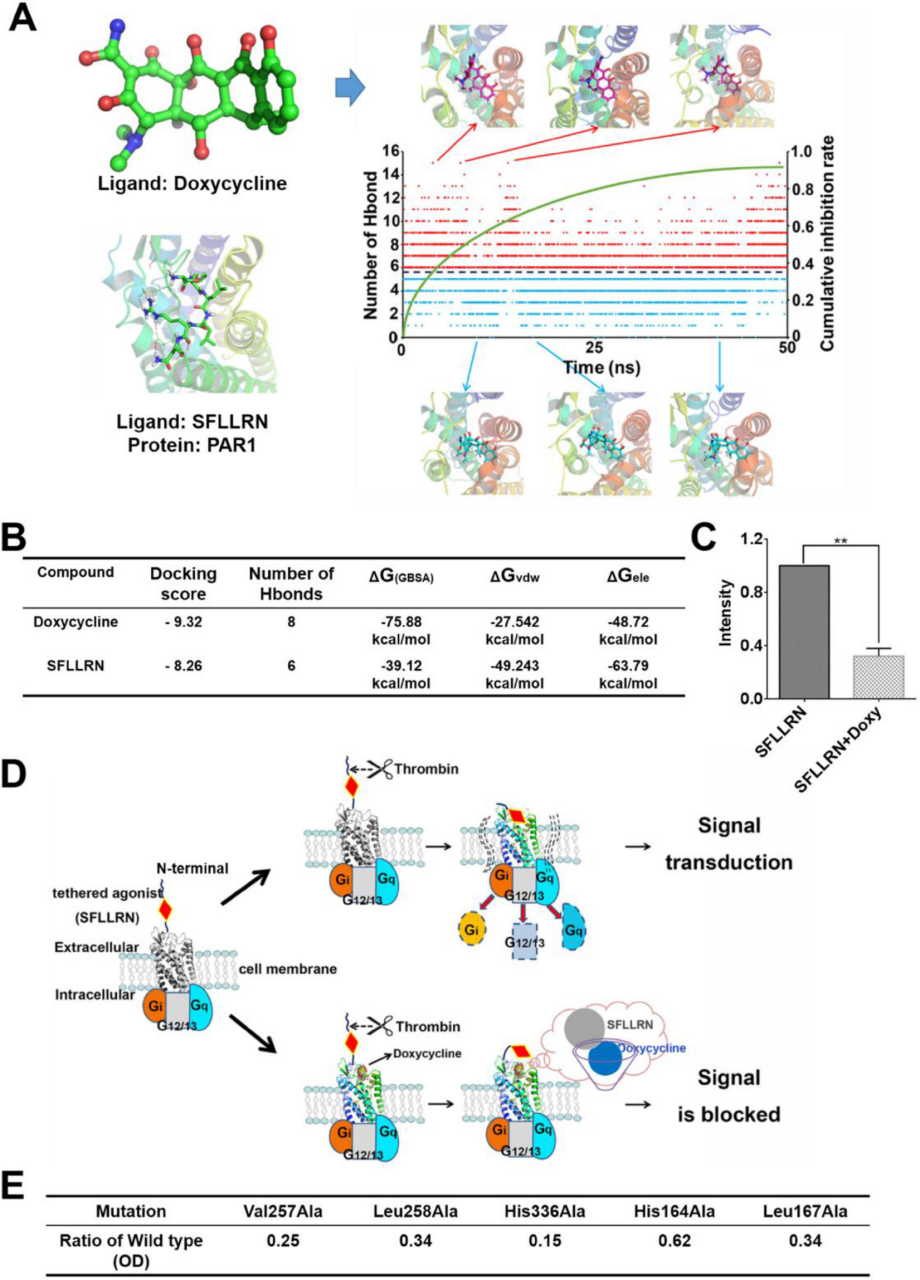
Molecular dynamic simulation and schematic diagram of the inhibitory mechanism showing direct interaction between doxycycline and PAR1 (**A**) Changes in the number of hydrogen bonds and typical conformations between PAR1 and doxycycline in our model system. Red dots indicate that the number of hydrogen bonds was greater than six; blue dots indicate that the number of hydrogen bonds was less than six. (**B**) Calculated results for the binding of doxycycline and SFLLRN to PAR1, indicating that doxycycline inhibition of PAR1 is more effective than activation of PAR1 by SFLLRN. (**C**) Calcium signaling assay for doxycycline and SFLLRN. These results reveal that doxycycline inhibits calcium influx caused by SFLLRN. (**D**) The mechanism underlying doxycycline inhibition of PAR1. Doxycycline occupies the active site of PAR1 and prevents “tethered agonist” activation, ultimately blocking signal transduction. (**E**) Mutation of PAR1 reveal key residues involved in doxycycline binding, which is consistent with the MD results. Results were obtained from three independent experiments, each performed in triplicate, and the error bars represent the standard deviation (*P < 0.05, ^*^P < 0.01). Data are represented as mean ± SEM.

To identify key amino acids of PAR1 that binding with doxycycline *in vivo*, we calculated the hydrogen bond interactions between the ligand and the receptor (Figure 3A). The hydrogen bond interactions of key residues are listed in Supplementary Table 2. Based on the molecular dynamic simulation results, the important residues involved His164, Leu167, Asp165, His245 and Tyr259. Six hydrogen bond formed during “tethered agonist” binding to PAR1 [17]. Normally, agonists and antagonists competitively bind to the active site of PAR1. During doxycycline binding to PAR1, a situation in which the number of hydrogen bond interactions was greater than 6, the antagonistic effect of doxycycline was greater than the activating effects of the agonist, which results in the suppression of PAR1. Consistent with the importance of hydrogen bonding, PAR1 remained activated when fewer than 6 hydrogen bond interactions were present between doxycycline and PAR1. The green curve shows that as the simulation time increased, the cumulative inhibition rate gradually increased. The results of the comparison between doxycycline and SFLLRN are presented in Figure 3B and show that doxycycline inhibition of PAR1 was more effective than PAR1 activation by SFLLRN. The calcium flow assay results were consistent with this calculation (Figure 3C). Doxycycline markedly inhibited 68% of calcium signaling activated by SFLLRN. Doxycycline occupies the active site of PAR1 and interacts with key amino acids thus preventing “tethered agonist (SFLLRN)” activation of the receptor (Figure 3D). Mutagenesis studies suggest that the mutation of residues involved in doxycycline binding, including Val257Ala, Leu258Ala, His336Ala, His164Ala, and Leu167Ala, markedly reduces PAR1 inhibition by doxycycline. The PAR1 His336Ala mutation impaired doxycycline binding affinity by more than 85% (Figure 3E). His336 is a key amino acid in the formation of hydrogen bond interactions with tetracyclines. Based on the above analysis, doxycycline inhibits PAR1 and thus prevents signal transduction.

### Principal component analysis of multi-omics data reveals that doxycycline targets the PAR1 pathway

**Figure 4:**
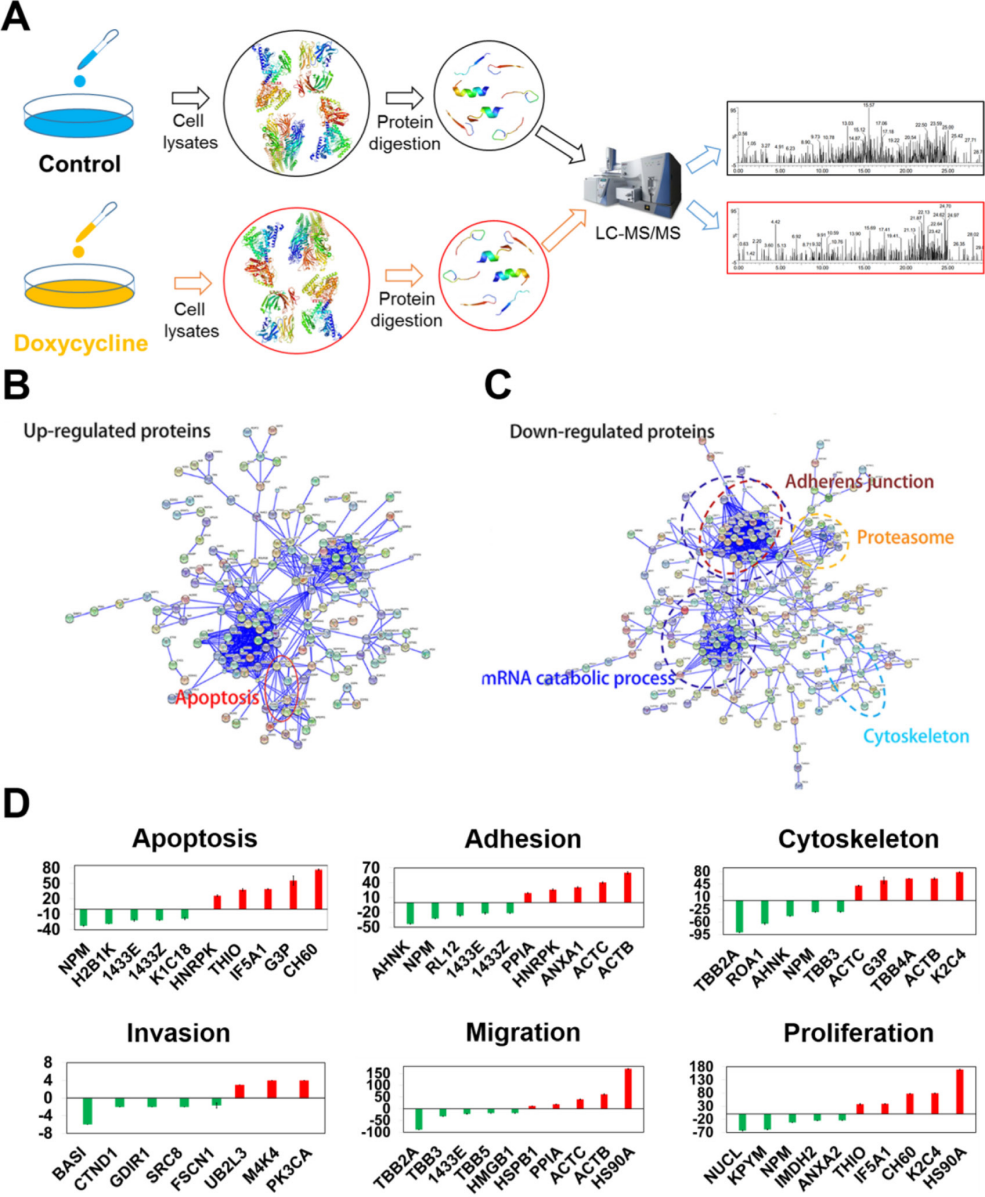
Differentially expressed proteins evaluated using multi-dimensional liquid chromatography-tandem mass spectrometry revealed key biological functions influenced by doxycycline Among the 231 differentially expressed proteins, 101 were increased, and 130 were decreased. (**A**) Workflow of the proteomics analysis. (**B**, **C**). Protein-protein interaction networks of differentially expressed proteins. Significantly changed proteins correlated with apoptosis, adherens junctions, the proteasome, mRNA catabolic processes and the cytoskeleton. (**D**) Pathways perturbed in a doxycycline-dependent manner were associated with apoptosis, adhesion, the cytoskeleton, migration, proliferation and invasion. Results were obtained from three independent experiments, each performed in triplicate, and the error bars represent the standard deviation (*P < 0.05, ^*^P < 0.01). Data are represented as mean ± SEM.

Proteomics assays were conducted to assess the effects of doxycycline on cancer cells. As shown in Figure 4A, the cells were lysed and digested, and differentially expressed proteins were analyzed by LC-MS/MS. STRING database was selected to examine several types of interactions between the control and doxycycline groups (Figure 4B and 4C) [18]. These results showed that many functions are influenced by doxycycline. The results of the protein-protein interaction network data showed that doxycycline enhances protein complex disassembly, mRNA splicing via the spliceosome and apoptosis. We also found groups of interacting proteins involved in the inhibition of adherens junctions, proteasome, mRNA catabolic processes and cytoskeleton dynamics in cells. Further analysis revealed a wide range of doxycycline-dependent effects in apoptosis, adhesion, cytoskeletal maintenance, migration, proliferation and invasion (Figure 4D). These results have been validated by several previous studies reporting on the anti-cancer effects of doxycycline.

**Figure 5:**
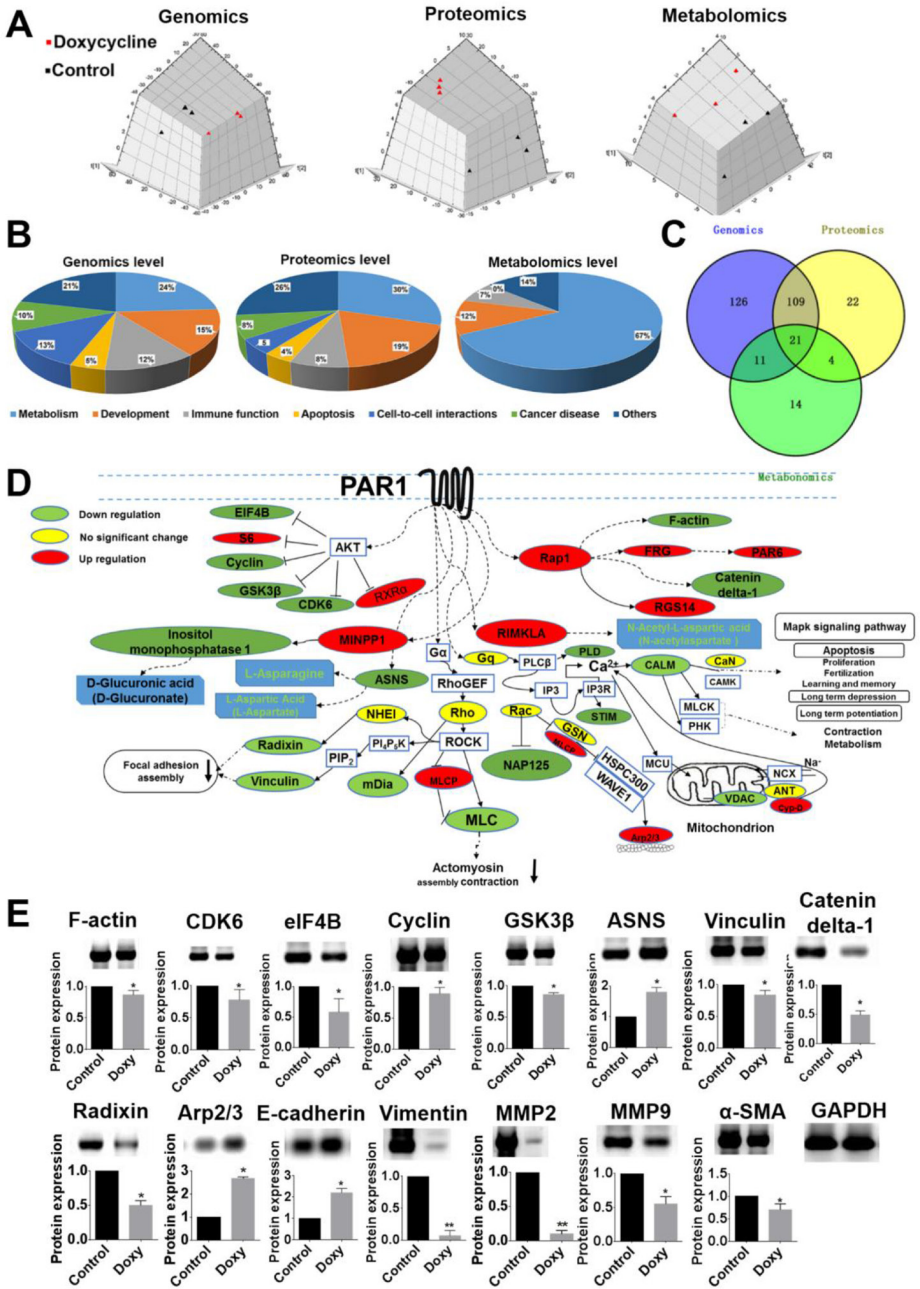
Multi-omics data analysis reveal that doxycycline inhibits PAR1-regulated signaling pathways (**A**) PCA analysis of genomics, proteomics and metabolomics. These results revealed a significant separation of the clusters between the control and doxycycline groups. (**B**) Statistical analysis of the pathways related to metabolism, cell growth, inflammation and apoptosis. (**C**) Unique and mutual pathways identified based on genomics, proteomics and metabolomics. (**D**) Proteins in the PAR1-regulated pathway are shown in green (down-regulated), red (up-regulated) or yellow (no significant change). The pathways that were influenced included those involved in actin stress fiber polymerization, actomyosin assembly for contraction, focal adhesion and the MAPK signaling pathway. (**E**) Western blotting was used to confirm the differential expression of proteins in the PAR1 pathway. Results were obtained from three independent experiments, each performed in triplicate, and the error bars represent the standard deviation (*P < 0.05, ^*^P < 0.01). Data are represented as mean ± SEM. See also Supplementary Figure 3.

To identify pathways in cancer cell lines that are inhibited by doxycycline, multi-omics level analyses were performed. As shown in Figure 5A, PCA analysis of the three omics revealed a significant separation of clusters between the control (black points) and doxycycline groups (red points). Among the differentially expressed genes, proteins and metabolites, the majority of influenced pathways were related to metabolism, cell development, immune function, apoptosis and cell-cell interactions (Figure 5B). Based on the multi-omics analysis, the main pathways that were influenced by doxycycline were identified, and the intersection of the three omics is shown in a Venn diagram (Figure 5C). The overlapping pathways identified by the three omics indicated a PAR1-regulated signal transduction cascade. Figure 5D provides a summary of the information related to the PAR1 pathway; the proteins in the PAR1 pathway that are differentially regulated by doxycycline treatment and verified by LC-MS/MS are shown in green (down-regulated), red (up-regulated) or yellow (no significant change) [19]. The main pathways are associated with focal adhesion assembly, actin polymerization in stress fibers, actomyosin assembly for contraction, actin stabilization, MAPK signaling, apoptosis and metabolism, all of which are associated with PAR1. Focal adhesion assembly as a locus of signal transduction activity plays a critical role in regulating signal transduction and viability processes in tumor cells. As shown in Figure 5D, the function of actin polymerization in stress fibers was inhibited. Stress fibers function in contraction and play an important role in cell morphogenesis, cell differentiation and tissue formation, and SEM analysis revealed similar results. Gene ontology (GO) analysis also revealed that the cancer cells were strongly influenced by the inhibit effect of doxycycline on PAR1, and various PAR1 signaling pathways and functions were inhibited (Supplementary Figure 3). All these analyses suggest that doxycycline targets PAR1, which hinders the progression of cancer cells. Western blot analysis was used to confirm the expression levels of key proteins and EMT markers in cancer cells (Figure 5E).

### Doxycycline specifically inhibits PAR1-positive tumor cells *in vitro* and *in vivo*

**Figure 6:**
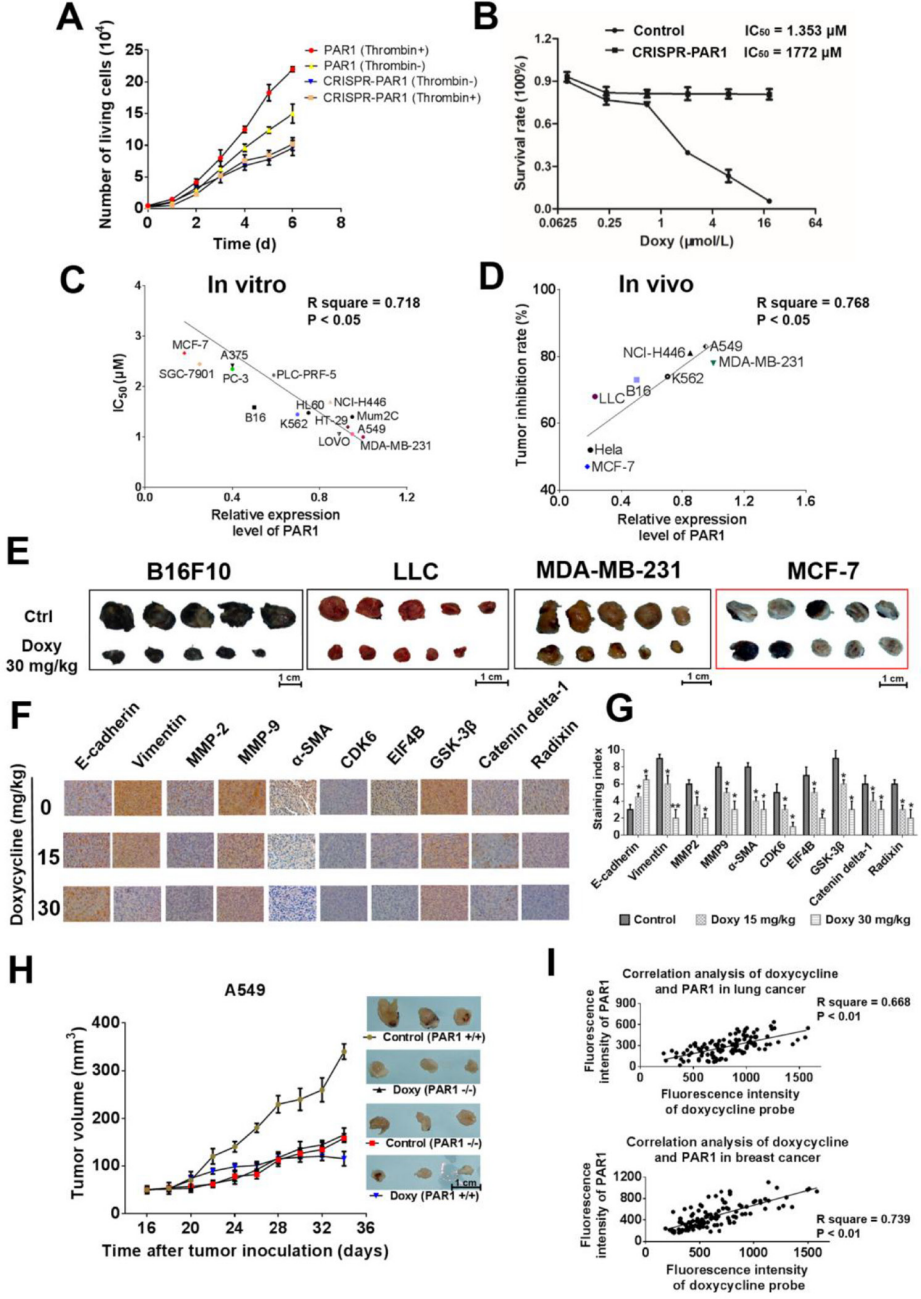
Doxycycline shows stronger inhibition and anti-tumor activity on cells with higher PAR1 expression (**A**, **B**) Loss of sensitivity to doxycycline in PAR1 knockdown cells. (**C**) Liner regression analysis of the doxycycline inhibition rate and the PAR1 expression level. These results showed a positive correlation between the two indexes. (**D**) Regression analysis of the doxycycline anti-tumor rate and PAR1 expression levels; the correlation coefficient was 0.768. (**E**) Doxycycline treatment inhibits xenograft growth in numerous models with dose of 15 and 30mg/kg. (**F**) Immunohistochemical staining to identify EMT biomarkers and doxycycline inhibited proteins in treated and untreated cells. Doxycycline-treated cells display stronger E-cadherin and α-SMA staining but reduced vimentin, MMP-2 and MMP-9 staining; the expression levels of other proteins were identical to those observed by western blotting. (**G**) Staining indexes of EMT biomarkers and doxycycline inhibited proteins. (**H**) Tumor volume and sensitivity to doxycycline (30 mg/kg) in control (PAR1^+^) and PAR1 knockdown (PAR1^-^) cell lines. These results suggest that PAR1^-^ cell lines lost their sensitivity to doxycycline. (**I**) Co-staining regression analysis of doxycycline and PAR1 in human tumor samples. These results show a clear positive correlation between the fluorescence intensity of doxycycline and PAR1. The regression analysis of the doxycycline staining intensity and pathological grading also showed a positive correlation. Results were obtained from three independent experiments, each performed in triplicate, and the error bars represent the standard deviation (*P < 0.05, ^*^P < 0.01). Data are represented as mean ± SEM. See also Supplementary Figure 4 and Supplementary Figure 5.

In the present study, tetracyclines, especially doxycycline, inhibit calcium influx caused by thrombin-mediated activation of PAR1, which results in decreased inhibition efficacy upon PAR1 knockdown (Figure 6A and 6B). Doxycycline exhibited highly selective inhibition of cancer cells with high expression levels of PAR1, and regression analysis revealed that the correlation coefficient between the IC_50_ and the expression level of PAR1 was 0.718 (*P* < 0.001) (Figure 6C and Supplementary Figure 4). Doxycycline also had higher anti-tumor activity toward cells with higher PAR1 expression in a xenograft model, with a correlation coefficient of 0.768 (*P* = 0.0043). Compared with the untreated control group, the tumor volume was significantly decreased after doxycycline treatment. Moreover, the sensitivity of tumors to doxycycline was associated with PAR1 expression in various cell lines (Figure 6E). For example, the minimally invasive MCF-7 cells, which express low levels of PAR1, were relatively insensitive to doxycycline inhibition [9] (See Supplementary Figure 5). These *in vivo* results strongly indicate that doxycycline targets PAR1 to inhibit tumor growth. Figure 6G shows immunohistochemical staining for EMT markers, invasive biomarkers and doxycycline inhibited proteins. Based on the immunohistochemical staining results, compared with the untreated doxycycline groups, E-cadherin and α-SMA exhibited high expression levels, whereas vimentin, MMP2 and MMP9 exhibited low expression in tumor tissue. The expression levels of other proteins were similar based on the three omics analyses and western blot analysis (Figure 6F and 6G). In A549 cells in which PAR1 was knocked down, the tumor formation rate and degree of malignancy were reduced compared with those of wild-type cells, and PAR1-deficient cells showed a loss of sensitivity to doxycycline (Figure 6H).

Two hundred and twenty-eight cases of pathological lung cancer specimens and 150 cases of pathological breast cancer specimens were used to assess the co-localization of doxycycline and PAR1. The staining intensity of doxycycline and PAR1 varied in different samples, but the fluorescence intensity of doxycycline and PAR1 expression showed a clear positive correlation (Figure 6I). These results show that this method can be applied clinically to screen for patients who are sensitive to doxycycline treatment.

## DISCUSSION

Activated PAR1 promote metastatic and invasive processes in various cancers [20, 21]. High expression levels of PAR1 were detected in various cancer cell lines, including MDA-MB-231, A549, LLC, PC-7 and HT-29 [22, 23]. Abundant experimental data and clinical investigations have suggested that PAR1 is an important target for cancer therapy [24, 25]. Here, we show that the inhibitory effects of doxycycline on cancer cells are mediated through PAR1.

In a previous study, doxycycline was found to inhibit angiogenesis in chick chorioallantoic membrane (CAM) samples by repressing the activity of matrix metalloproteinases (MMPs) [26, 27]. Until now, doxycycline was still considered an anti-angiogenesis drug that functions through the inhibition of MMPs. However, the evidence was not strong enough to confirm that doxycycline could directly inhibit MMPs. Although fourteen clinical trials investigating the application of doxycycline in cancer therapy are currently being conducted through the FDA, the therapeutic effects of doxycycline are inferior to those of targeted drugs (See Supplementary Table 3).

Patients with doxycycline-sensitive tumors could show greater benefit if doxycycline is used as a targeted therapeutic in clinical applications. The effects of doxycycline are reflected by its multi-target profile, and thus the major problem has been the identification of its main target. In this study, we used “click” chemistry to identify the target of doxycycline. Although non-specific bands were observed, coomassie staining, western blotting and mass spectrometry strongly confirmed the ion between doxycycline and PAR1. Interestingly, doxy-yne cannot capture internalized PAR1 when PAR1 is combined with its natural ligand and internalized. This finding indicates that doxycycline can interact with only PAR1 located on the membrane and that doxycycline has the same binding site as a natural ligand of PAR1 (Supplementary Figure 6).

Calcium ions are the second messenger of the PAR1 signaling pathway. The detection of calcium influx is a standard model to screen for inhibitors of PAR1. The results showed that tetracyclines, especially doxycycline, inhibit the calcium influx elicited by PAR1. Compared with a reported PAR1 inhibitor (Vorapaxar), which is a virtually irreversible PAR1 antagonist, the inhibition ability of doxycycline was 3-fold less [28]. Vorapaxar and doxycycline exhibited the same binding mode at the active site of PAR1, with docking scores of 8.2 and 6.7, respectively. Vorapaxar has been approved by the FDA, but this drug is associated with an increased risk of bleeding and calcium metabolic abnormalities. These side effects are similar to those of doxycycline when used in antibiotic therapy.

Multiple signal transduction pathways are inhibited by PAR1, including cell growth, differentiation, migration, and PAR1 especially influences the functions of endothelial cells. Endothelial cells originate from the mesoderm and have a similar origin and similar biomarkers to those of melanoma, sarcoma, neurogenic tumors (oat cell cancer) and triple-negative breast cancer [29]. Doxycycline was found to inhibit cell progression and tumor growth of cells with high PAR1 expression both *in vitro* and *in vivo*. Using CRISPR, we knocked down PAR1 expression and found that the inhibitory activity of doxycycline toward PAR1 was lost. These results strongly indicate that doxycycline directly targets PAR1 to suppress tumor progression in a dose-dependent manner, and these results provide an explanation as to why doxycycline exerts anti-vascular effects, which includes the inhibition of MMPs.

Based on immunohistochemical staining results in tumor tissues, the difference in protein expression after doxycycline treatment was highly consistent with that of PAR1 repression. In A549 cells in which PAR1 was knocked down, the tumor-formation rate and degree of malignancy were reduced compared with PAR1 wild-type cell lines. We used 228 pathological lung cancer specimens and 150 pathological breast cancer specimens to perform fluorescence co-localization analysis of doxycycline and PAR1. The fluorescence intensity of doxycycline and PAR1 displayed a clear positive correlation. These results indicate that paraffin-embedded tumor tissues could be used to evaluate drug sensitivity. Drugs can show increased binding to the pockets of proteins after formaldehyde fixation; however, doxycycline binding to the pocket of PAR1 was not seriously suppressed by fixation, dehydration, embedding or sectioning of the tumor samples. In clinical trials, this method can provide evidence for patients with tumors that are sensitive to drugs such as doxycycline. In an animal tumor model, samples that stained positively for both doxycycline and PAR1 also showed a strong sensitivity to doxycycline.

In summary, the current work indicates that doxycycline directly targets PAR1 and reveals that the anti-tumor mechanism of doxycycline is mediated through PAR1. According to the results of our molecular experiments, our observed anti-tumor effects upon doxycycline treatment and our pathological analysis experiments, we further identified and characterized a critical role for PAR1 in tumor cells, and we show evidence that doxycycline should be treated as a targeted drug for clinical applications. Our results support the use of doxycycline as a lead compound for PAR1 inhibition and pave the way for ongoing anti-tumor doxycycline therapy.

## MATERIALS AND METHODS

### General information

Doxycycline-HCl powder (purity 99.99%) was obtained from KAIFENG YUGANG PHARMACEUTICAL CO., LTD (Henan, China) and was dissolved in sterile 0.9% NaCl immediately before use. The click chemistry catalyst ligand Tris [(1-benzyl-1H- 1,2,3-triazol-4-yl) methyl] amine (TBTA) and Tris (2-carboxyethyl) phosphine (TCEP) were purchased from Sigma-Aldrich. The antibody against PAR1 was purchased from Abcam. All mutant assays were performed according to the protocols provided by the manufacturer. The QuickChange site-directed mutagenesis kit was used to generate mutant derivatives from the corresponding wild-type constructs.

### Cell culture

The human carcinoma cell lines A549, HEK-293, PLC-PRF-5, H446, LLC, MCF-7, MDA-MB-231, HCT-116 and BL16F10 were obtained from KeyGen Biotech (Nanjing, China). The cells were cultured in RPMI-1640 medium (Hyclone, USA) supplemented with 10% fetal bovine serum (Hyclone, USA) and antibiotics (50 units/mL penicillin and 50 μg/mL streptomycin). All cultures were maintained at 37°C in a humidified atmosphere containing 5% CO_2_.

### Synthesis of the doxycycline probe doxy-yne

Doxy-yne synthesis was performed using commercially available doxycycline as the raw material. The terminal alkyne-containing doxy-yne probe was obtained by coupling doxycycline to 5-hexynoic acid and 2-(3-but-3-ynyl-3H-diazirin-3-yl)-ethanol. The fluorescent group rhodamine-N_3_ and biotin-N_3_ were synthesized using previously published procedures.

### MTT assay

Exponentially growing cells were seeded at 5×10^4^ cells/mL in 96-well plates. Doxycycline was administered at various concentrations, and then the plates were incubated for 48 h before analysis. The optical density (OD) values were determined by measuring the absorption at 570 nm using a microplate reader (Multiskan^™^ FC, Thermo Scientific, Waltham, MA, USA).

### Labeling of A549 cells

The A549 cell line was seeded in 60-mm dishes at 80% confluence, after which doxy-yne was added to the cells at the desired final concentration. After incubation for 4 h at 37°C with 5% CO_2_, the cells were UV-irradiated (365 nm) for 30 min. After cell lysis, 0.1 mM TBTA, 1 mM TCEP, 0.1 mM rhodamine-N_3_ and 1 mM CuSO_4_ were added to the samples. The reaction was then further incubated for 2 h, and the proteins were precipitated with acetone and further processed by SDS-PAGE and in-gel fluorescence scanning. Fluorescent strips were cut out and analyzed by mass spectrometry.

### Doxycycline probe pull-down assay

Positive pull-down: The cells were lysed and protein A/G-PAR1 antibody was added to the lysates. The lysates were further incubated with doxycycline labeled with a fluorescent probe. Finally, the complex was measured by ultraviolet spectrophotometry. Negative pull-down: Doxycycline labeled with biotin was incubated with streptavidin magnespheres, and then streptavidin magnesphere-Doxy was obtained. This complex was added to the cell lysates. Using an ELISA assay, PAR1 was detected using a microplate reader. All steps were performed at 4°C.

### Biacore assay

SPR experiments were performed using a Biacore 3000 instrument (GE Healthcare, Piscataway, NJ, USA). PAR1 was purified from 293-PAR1 cell lines using antibody-containing immunomagnetic beads. PAR1 was immobilized on CM5 sensor chips using the Biacore Amini Coupling Kit according to the manufacturer’s instructions. Doxycycline was diluted in running buffer and then injected into PAR1-immobilized CM5 sensor chips at concentrations of 20 μM, 40 μM and 80 μM. The surface of the control chip was prepared in the same manner and was used for data correction. Data analysis was performed using BIAevaluation software.

### Immunofluorescence staining and live cell imaging

Adherent cells were seeded in dishes, grown to 80% confluence and treated with 0.2 μM doxy-yne or DMSO for 5 h, followed by UV irradiation (365 nm) for 30 min. The cells were fixed for 15 min in 3.7% paraformaldehyde and washed twice with PBS, after which they were treated with 0.1% Triton X-100 for 10 min. Non-specific binding was blocked by incubation with 3% BSA for 30 min, followed by two more washes with PBS and treatment with a click chemistry reaction solution. The final concentrations of the catalyst and dye were as follows: 1 mM CuSO_4_, 1 mM TCEP, 100 μM TBTA and 10 μM rhodamine-N_3_ in PBS for 2 h at room temperature with shaking. For the co-localization experiments with PAR1, the primary antibody against PAR1 (1:100 dilution) was added for 1 h at room temperature, and then the cells were washed and incubated with fluorescein isothiocyanate (FITC)-conjugated anti-rabbit IgG (1:500) for 1 h.

### Sample preparation for single molecule image co-localization using super-resolution microscopy

A549 cells were seeded in 35-mm N-STORM super-resolution microscope dishes and were grown to 60% confluency. Next, 0.2 μM doxy-yne was added to the cells and incubated for 4 h at 37°C in the presence of 5% CO_2_, followed by UV irradiation (365 nm) for 30 min. The cells were then fixed and permeabilized and 0.1 mM TBTA, 1 mM TCEP, 0.1 mM Alexa 647-conjugated azide (Thermo Fisher, USA) and 1 mM CuSO_4_ were added to the cells for 2 h for the click reaction. After the click reaction, the cells were incubated with rabbit polyclonal anti-PAR1 (CST, USA) in a humidified chamber overnight at 4°C and then were incubated with Cy3B-conjugated goat anti-rabbit secondary antibody for 2 h at room temperature, followed by seal slicing. The N-STORM super-resolution microscope (Nikon, Japan) was used to measure doxycycline and PAR1 co-localization on the nanoscale level.

### Scanning electron microscopy

Cells grown on climbing films (WHB, China) were treated with or without doxycycline (0.2 or 1.6 μM). After 24 h of incubation, the cells were fixed and dehydrated in acetone/isoamyl acetate (1:1), dried with a gradient concentration of acetonitrile and finally coated with gold. Images of the cells were obtained using a scanning electron microscope (LEO 1530 VP, Germany).

### Molecular docking

Molecular docking was performed using Sybyl X1.1 software. The crystal structure of PAR1 in a complex with Vorapaxar was downloaded from the PDB database (PDB code 3VW7). The Vorapaxar ligand of PAR1 was extracted from the crystal structure as the location center of docking.

### Inhibition of calcium influx assay

HEK-293 cells stably transfected with PAR1 were seeded into blank-walled 384-well plates. After 12 h of incubation to allow the cells to adhere to the plate, the culture medium was aspirated, and the cells were stimulated with thrombin (1 U/mL) or seroperitoneum obtained from mice that had been injected with *Escherichia coli* and then mixed with doxycycline and other tetracyclines. The Calcium 5 kit was used for the loading dye, and a FlexStation III device (Molecular Devices, SMP500-19897-MILX) was used to evaluate changes in fluorescence.

### CRISPR/Cas9 system knockdown of PAR1

The clustered regularly interspaced short palindromic repeats (CRISPR)-associated nuclease 9 (CRISPR/Cas9) system was used to knockdown PAR1 in A549 and HEK-293 cells. One single guide RNA (sgRNA) was designed to target the upstream genomic sequence of the PAR1 and the downstream sequence. Knockdown cells were evaluated by PCR and DNA sequencing. Monoclonal cells lacking PAR1 were selected for further experiments.

### Matrigel invasion assay

Using 24-transwell plates (Corning, America), a total of 5 × 10^4^ cells were placed on the top chamber inserts coated with Matrigel (BD Biosciences, New Jersey, America). After 48 h of incubation, the cells were stained with 0.1% crystal violet and counted under a microscope.

### Wound healing assay

A549 and A549 PAR1(-/-) cells cultures grown to 90% confluency in 24-well culture plates were wounded and then incubated with doxycycline and thrombin respectively. Then, cell membrane red fluorescent dye (DiD, KeyGEN BioTECH, Nan Jing, China) were incubated cells and the wounds were photographed at 0, 24 and 48 h using a fluorescence microscope in 561 nm (Nikon, Japan).

### Three-dimensional culture assay

Cells were seeded in a 96-well plate precoated with Matrigel. The cells were then treated with thrombin or thrombin-doxycycline separately for 24 h. Images of the cells were obtained using a light microscope (Nikon, Japan).

### Molecular dynamic simulations

The Pmemd module of the Amber 14 package was chosen to perform all energy minimizations and molecular dynamic simulations. The entire MD system was heated to 310 K to simulate the temperature of a normal physiological reaction. We used periodic boundary conditions in the NPT ensemble and the SHAKE algorithm to constrain all covalent bonds containing hydrogen atoms. For non-bonded interactions, the cutoff values were set at 10 Å. Finally, the root-mean-square deviation (RMSD) of the initial structure from the simulated positions was used to evaluate the stability of the entire simulation.

### Multi-omics analysis

A549 cells were untreated or treated with doxycycline for 24 h, after which the cells were lysed to isolate RNA, protein and metabolites. Proteins were digested with trypsin for 20 h at 37°C. The sample was evaporated and resuspended in water/0.1% formic acid for LC-MS/MS analysis. The experiment was repeated three times. The largest dataset selected was Swiss-Prot, Human, which included 20,258 protein sequences. Data analysis was performed to reveal critical pathways. Gene ontology (GO) analysis was used to detect doxycycline-induced changes in molecular functions, biological processes and cellular components. The STRING database was used to analyze protein-protein interactions, and KEGG pathway analysis revealed that various signaling pathways were inhibited.

### Murine xenograft model

Six-week-old female NIH BALB/c-null and C57BL/6J mice were maintained in specific pathogen-free animal facilities at Tianjin International Joint Academy of Biomedicine. The mice were divided into 3 groups randomly (*n* = 10/group). When the volume of the tumor reached approximately 50 mm^3^, the model mice were treated with 30 or 15 mg/kg of doxycycline or saline as a control. The tumors were evaluated using the following formula: V = ab^2^/2 (a = length of tumor, b = width of tumor). All mice were euthanized after six weeks of treatments, and tumor, lung and liver tissues were fixed for further immunopathological studies. An equivalent number of mice were grouped and injected with 1 × 10^7^ cells via the caudal vein to record the survival time of each mouse.

### Immunohistochemical analysis

The tissues were incubated with xylene for deparaffinization and decreasing concentrations of ethanol for rehydration. Next, 3% hydrogen peroxide was applied to block endogenous peroxidase activity. The microwave antigen retrieval technique was utilized for antigen retrieval. After blocking, the samples were incubated with primary antibodies overnight at 4°C: rabbit polyclonal anti-PAR1 (Affinity, ready-to-use), rabbit polyclonal anti-vimentin (Affinity, 1:50 dilution), rabbit polyclonal anti-MMP-2 (Zhongshan, ready-to-use) and rabbit polyclonal anti-MMP9 (Abcam, 1:100 dilution). Tumor cells with brown-stained cytoplasm, nuclei or membranes were considered positive and were scored as follows: none (0), weak brown (1+), moderate brown (2+) and strong brown (3+). The percentage of stained tumor cells was divided into five classes: 0 for negative cells, 1 for 1–25%, 2 for 25–50%, 3 for 50–75% and 4 for > 75%.

### Patient samples

The study group consisted of 228 cases of pathological lung cancer specimens and 150 cases of pathological breast cancer specimens included in tissue microarrays, which were obtained from US Biomax and subjected to immunofluorescence co-localization analysis. Lung and breast cancer slides were assessed for the pathological diagnosis by two pathologists based on hematoxylin and eosin-stained sections. All samples were associated with detailed pathological and clinical data, including age, pathological stage, tumor diameter, nodal status and lymph node metastasis. This information was obtained from primary pathology reports. The histological types and grades of all tissue spots were reviewed and defined by more than two pathologists. All human tissues were collected using HIPAA- and IRB-compliant protocols, and all patients signed informed consent forms.

### Statistical analysis

All data are expressed as the means ± standard deviations (SD). After testing for normality and equal variance across the groups, intergroup differences were assessed using Student’s *t*-tests, ANOVA and multivariate statistical analysis. Every experiment was repeated three times. *P* < 0.05 was considered statistically significant.

## SUPPLEMENTARY MATERIALS FIGURES AND TABLES


