# Supplementary material for: Doxycycline directly targets PAR1 to suppress tumor progression
Source: Oncotarget. 2017 Feb 7;8(10):16829–42. doi: 10.18632/oncotarget.15166 (PMC5370004; doi:10.18632/oncotarget.15166)
Supplement: Supplementary file 1 [file oncotarget-08-16829-s001.pdf]

# Doxycycline directly targets PAR1 to suppress tumor progression

## Supplementary Materials

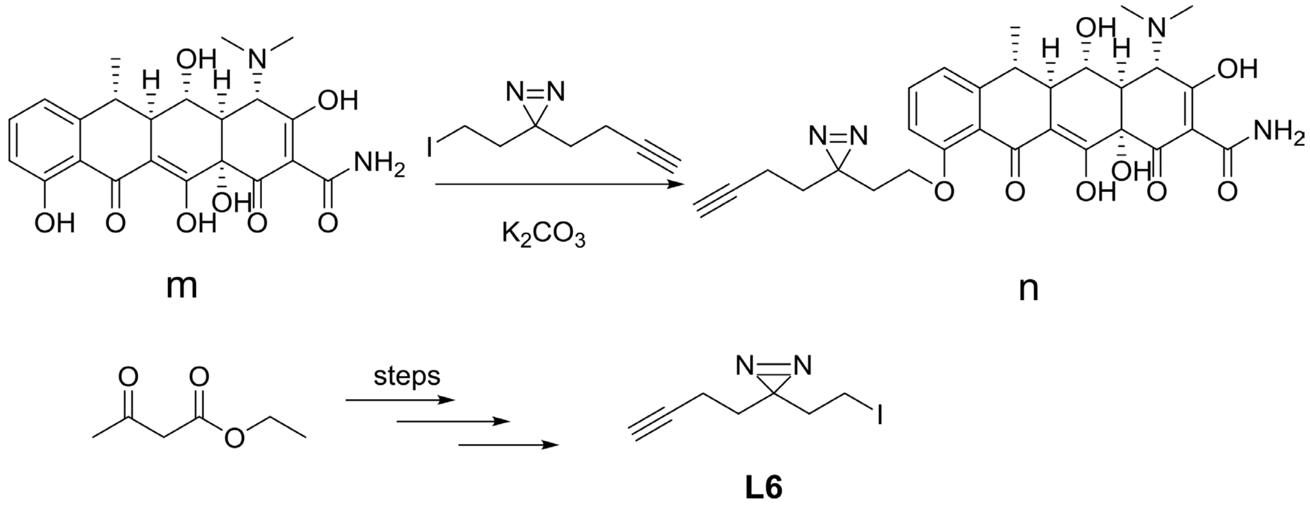


**Supplementary Figure 1: Related to Figure 1 Synthetic process and characterization result of doxycycline probe (Doxy-yne).** To a solution of (m) (94 mg, 0.183 mmol) in dry DMF (3 ml) was added L6 (50 mg, 0.2 mmol) and K2CO3 (50 mg, 0.369 mmol). The mixture was stirred under Ar overnight at 60°C. The reaction mixture was concentrated. The residue was purified by HPLC. 1H NMR (400 MHz, DMSO-d6): δ 7.56 (t, J = 8.0 Hz, 1H), 6.95 (d, J = 7.7 Hz, 1H), 6.90 (d, J = 8.4 Hz, 1H), 4.03 (m, J = 7.1 Hz, 1H), 3.45 (m, J = 7.0 Hz, 1H), 3.25 (t, J = 6.4 Hz, 2H), 2.92 – 2.80 (m, 8H), 2.56 (m, J =8.3 Hz, 1H), 2.02(m, 3H), 1.59 (t, J = 7.5 Hz, 2H), 1.48 (t, J = 3.6 Hz, 2H), 1.19 (d, J = 7.1 Hz, 3H). ESI-MS (m/z): 565.35 ([M+H+]).

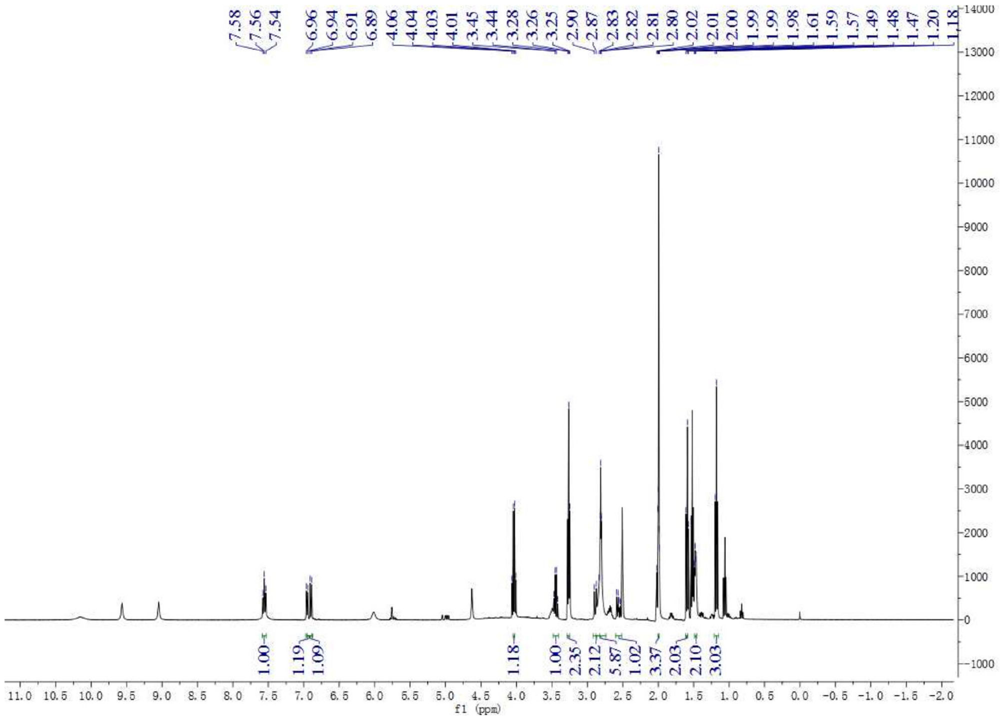


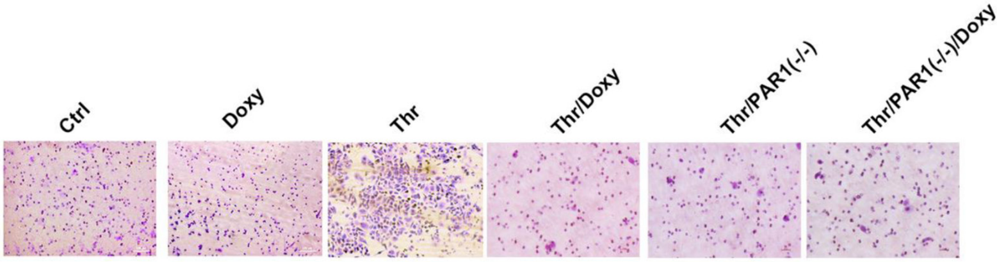


**Supplementary Figure 2: Related to Figure 2 Transwell assay of doxycycline on A549 cell lines.** Doxycycline inhibited the invasion of thrombin-stimulated cancer cells, and loss sensitivity to knock out PAR1 cells.

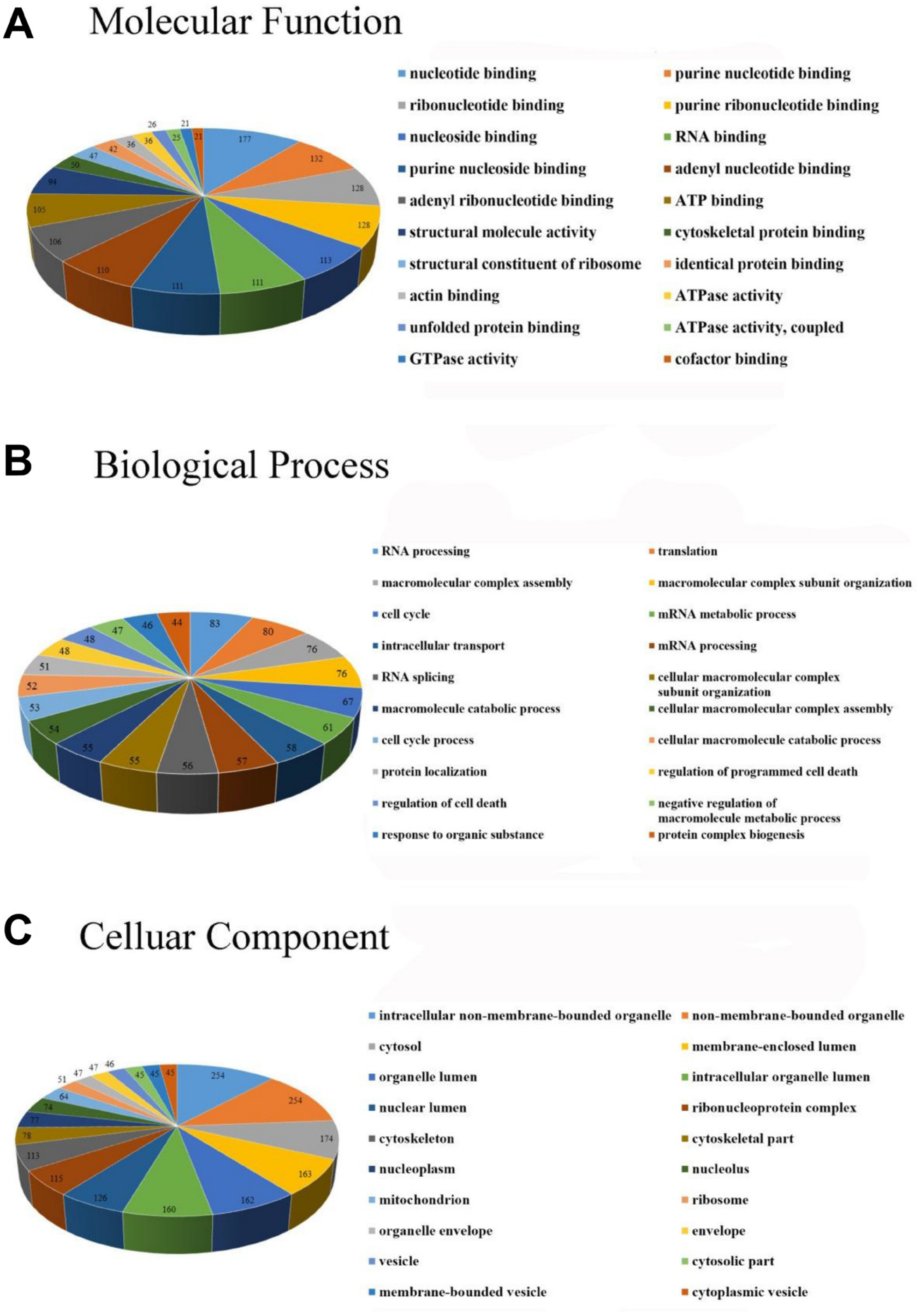


**Supplementary Figure 3: Related to Figure 4 GO analysis of doxycycline effect on cells.** (**A**) Classification of significantly differentially expressed proteins by gene ontology analysis including molecular function. Doxycycline influenced nucleotide binding, purine nucleotide binding, cytosol, vesicle, actin binding, cytoskeletal protein binding and ATP binding. Of interest, ATP binding involves energy metabolism, RNA binding influences the synthesis of proteins and the regulation of cell death, which have a marked effect on cell survival. Based on the above results, multiple biological processes that are essential for cell survival were influenced or inhibited by doxycycline. (**B**) Classification of significant differential expression proteins by gene ontology analysis including biological processes. According to the DAVID molecular function information, the top network function-based clusters were associated with RNA processing, translation, the cell cycle, mRNA metabolic processes, the regulation of programmed cell death, the response to organic substances and protein complex biogenesis. (**C**) Classification of significantly differentially expressed proteins by gene ontology analysis including cellular components. The analysis revealed that doxycycline stimulated cells produce a higher proportion of proteins that function in the following categories: non-membrane-bound organelles, intracellular non-membrane-bound organelles, cytosol, organelle lumen ribosome, ribosomes, and ribonucleoprotein complexes.

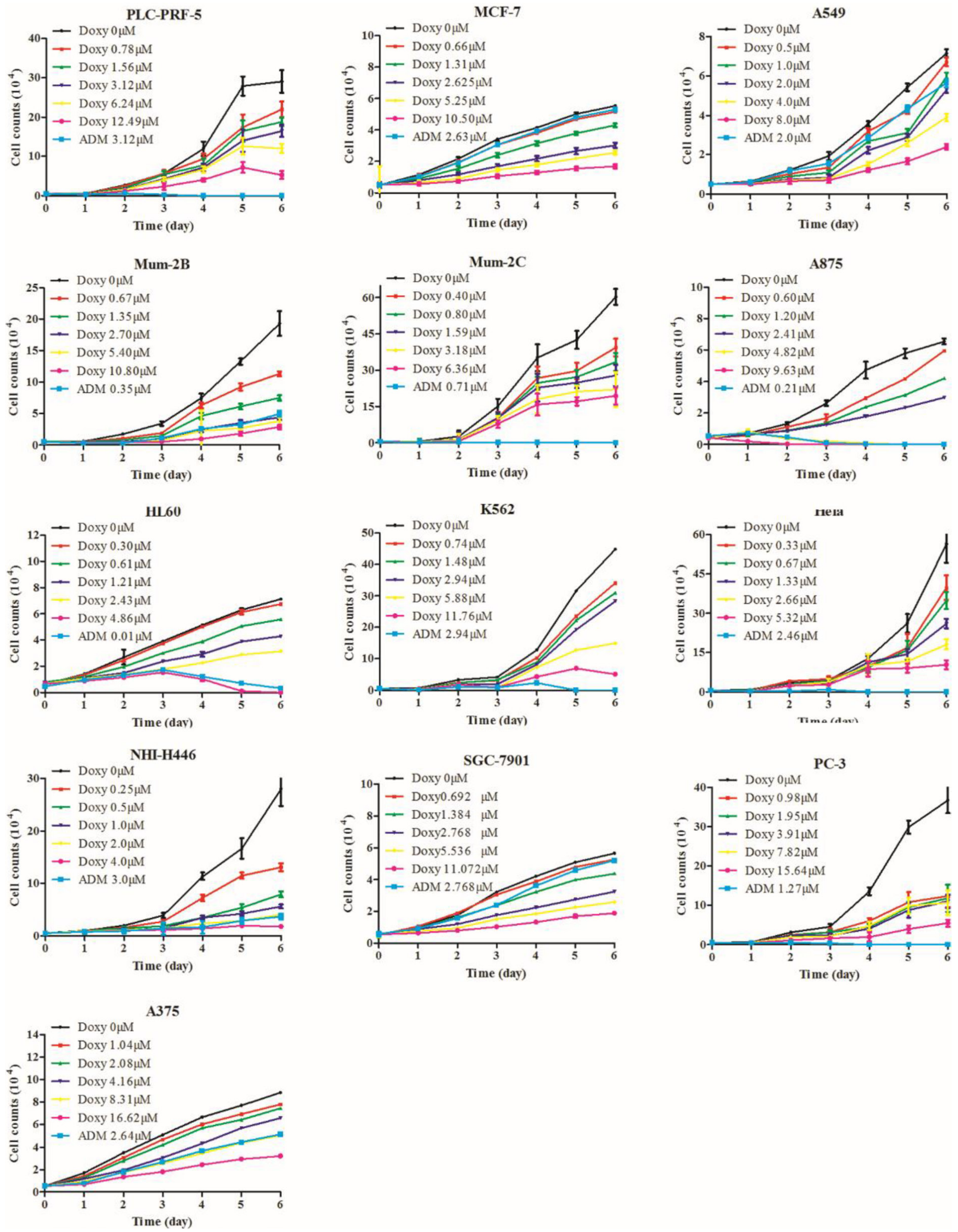


**Supplementary Figure 4: Related to Figure 6 Effect of doxycycline on the cell proliferation.** Growth curve method was used to measure the inhibition effect of doxycycline in different cancer cell lines.

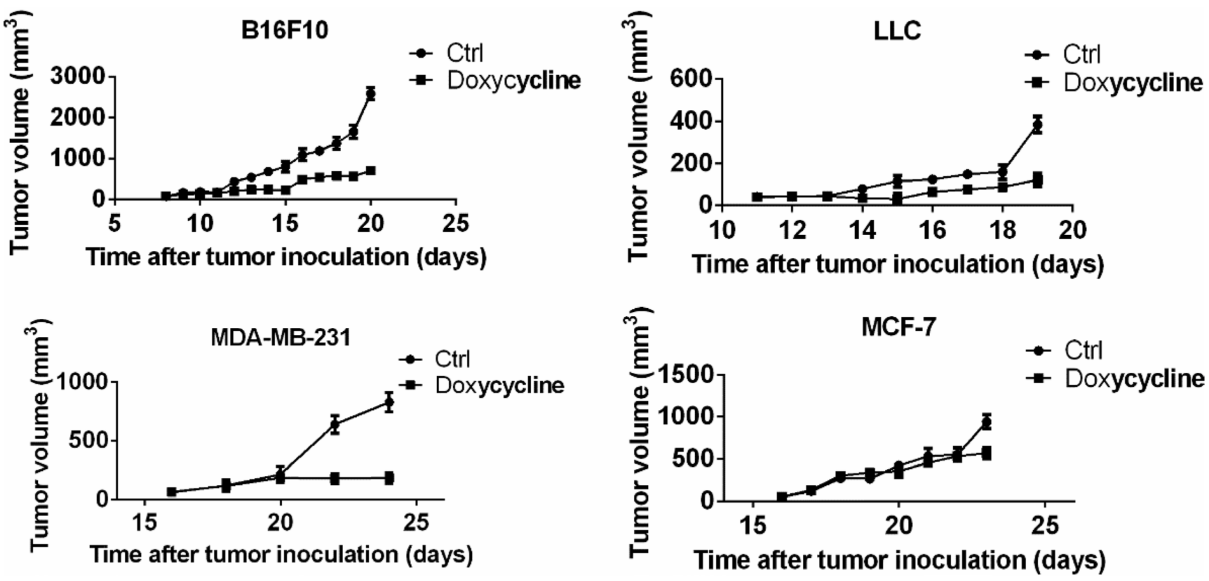


**Supplementary Figure 5: Related to Figure 6 Tumor growth curves of B16F10, LLC, MCF7 and MDA-MB-231 xenotransplanted tumor model treated with doxycycline.**

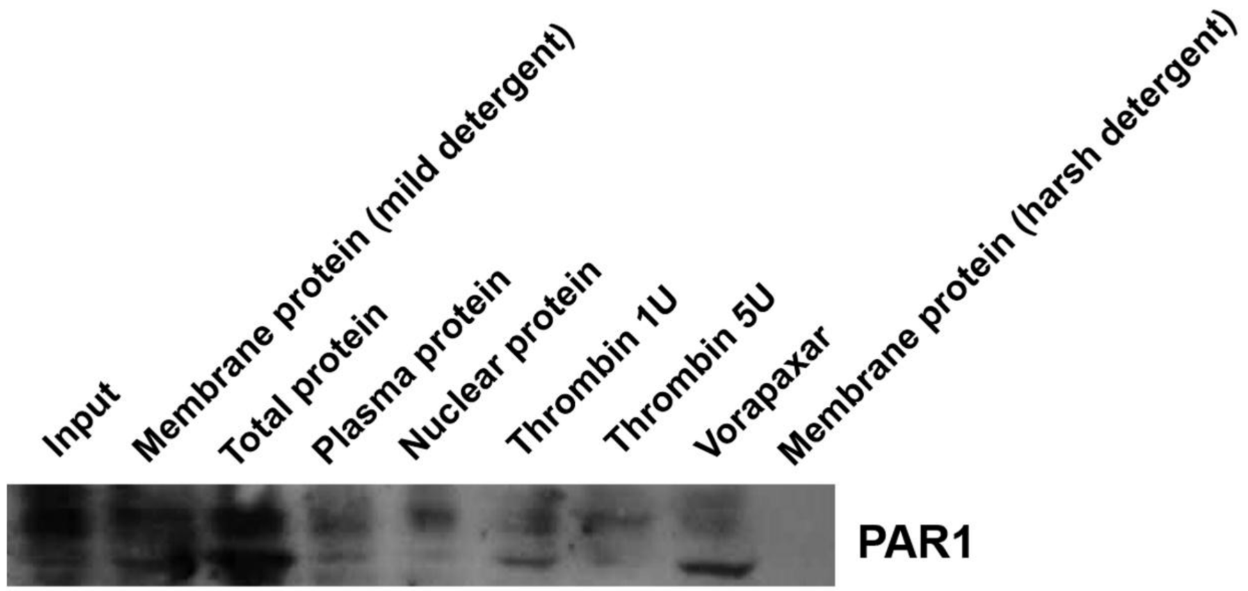


**Supplementary Figure 6: Western blot analysis of difference treatment of A549 cells.**

**Supplementary Table 1: Binding energy of tetracyclines binding into PAR1 active site**

| Compounds | $\Delta G_{Bind}$ | $\Delta G_{Coulomb}$ | $\Delta G_{Covalent}$ | $\Delta G_{Hbond}$ | $\Delta G_{Lipo}$ | $\Delta G_{vdW}$ |
|---|---|---|---|---|---|---|
| Doxycycline | −75.884 | −48.722 | 13.411 | −2.017 | −50.724 | −27.542 |
| Aureomycin | −72.831 | −52.635 | 16.141 | −1.331 | −46.552 | −40.644 |
| Penimepicycline | −62.027 | −93.767 | 2.072 | −0.974 | −36.586 | −36.935 |
| Oxytetracycline | −57.850 | −49.905 | 12.132 | −1.872 | −43.259 | −20.190 |
| Methacycline | −56.372 | −46.052 | 4.974 | −0.565 | −27.125 | −31.111 |
| Tigecycline | −52.741 | −29.953 | 7.117 | −0.395 | −38.666 | −44.98 |

**Supplementary Table 2: Key amino acids in the interaction of PAR1 with doxycycline**

| Acceptor | DonorH | Donor | Frames | Frac | AvgDist | AvgAng |
|---|---|---|---|---|---|---|
| HIE_164@O | Doxy_288@HO30 | Doxy_288@O30 | 1051.000 | 0.955 | 2.731 | 161.381 |
| Doxy_288@O26 | HIE_164@HE2 | HIE_164@NE2 | 654.000 | 0.594 | 2.893 | 147.833 |
| Doxy_288@O27 | LEU_167@H | LEU_167@N | 603.000 | 0.548 | 3.221 | 154.773 |
| Doxy_288@HO30 | ASP_165@HA | ASP_165@CA | 558.000 | 0.507 | 3.253 | 141.917 |
| Doxy_288@H8 | HIE_245@HB2 | HIE_245@CB | 464.000 | 0.421 | 3.261 | 147.192 |
| LEU_167@O | Doxy_288@H5 | Doxy_288@N1 | 460.000 | 0.418 | 2.975 | 146.564 |
| Doxy_288@O25 | TYR_259@HA | TYR_259@CA | 339.000 | 0.308 | 3.367 | 144.033 |

**Supplementary Table 3: Clinical trials of doxycycline for cancer therapy in FDA.**

See Supplementary_Table_3
